# Knowledge of Surgery-Related Adverse Effects Caused by Cigarette Smoking Among the Makkah Population, Saudi Arabia

**DOI:** 10.7759/cureus.99676

**Published:** 2025-12-19

**Authors:** Reham N Alsaud, Khalid H Alnafei, Abdullah S Alibrahim, Muath S Alshami, Rayan A Al Harthi, Ahmad H Tatwany, Abeer Shaker

**Affiliations:** 1 College of Medicine and Surgery, Umm Al-Qura University, Makkah, SAU; 2 Surgery, Royal Commission Yanbu Medical Center, Yanbu, SAU; 3 Pathology, Umm Al-Qura University, Makkah, SAU

**Keywords:** adverse effects, cigarette smoking, knowledge, saudi arabia, surgery

## Abstract

Background

Smoking is a common condition that poses a huge risk to public health. It is a high-risk factor for multiple diseases and has an association with postoperative complications. Fortunately, the risk of postoperative complications can be reduced preoperatively by smoking cessation. Previous studies emphasize the lack of knowledge regarding surgery-related adverse effects caused by cigarette smoking among the general population. In this study, we aim to evaluate the level of knowledge about surgery-related adverse effects of cigarette smoking in the Makkah region.

Methods

A descriptive cross-sectional study using an online validated questionnaire was carried out to assess the level of knowledge about the surgery-related adverse effects of smoking. between the 8th of March 2024 and the 8th of April 2024. The questionnaire was distributed among adults in the Makkah region.

Results

In this study, 404 out of 447 participants were included. Of the participants, 206 (51%) were females, and 198 (49%) were males. Regarding smoking, 343 participants were non-smokers (84.9%), and the majority, 378 (93.6%) in total, possessed knowledge about smoking-related surgical adverse effects. A significant association was found between the level of knowledge about smoking health risks and smoking status (P = 0.015, adjusted odds ratio (ORA) = 0.850 and 95% Cl for odds ratio (OR) 0.809-0.894). Most of the participants (84.2%) agreed that it is necessary to stop smoking after surgery (in addition to preoperative smoking cessation), while 14.4% did not know. Most participants could identify that the most common health risks due to smoking are lung cancer, esophageal cancer, and coronary artery disease.

Conclusion

This study revealed limited awareness of the surgical risks of cigarette smoking despite generally good knowledge of its overall health effects. Targeted educational and cessation programs, including preoperative counseling and accessible support services, are essential to address this gap in the Makkah population.

## Introduction

Smoking is a substantial public health issue worldwide. According to statistics from the World Health Organization, roughly one billion men and 250 million women smoke [1], with smoking being a major life-threatening factor causing 24% of all male deaths and 7% of all female deaths worldwide, and a risk factor for multiple diseases such as cancer, chronic obstructive pulmonary disease (COPD), and atherosclerosis. Sadly, smoking is quite prevalent in Saudi Arabia [2], with a range of 2.4-52.3% (median = 17.5%) reported for the prevalence of current smoking [3]. Extended exposure to smoke can lead to a rise in oxidant levels in plasma [4,5]. In addition to those who smoke actively, passive smokers are also in danger of contracting illnesses as a result of smoking [6].

There is an association between smoking and an increased rate of postoperative complications such as poor wound healing, respiratory complications, general infections, and neurological complications [7,8]. Fortunately, such complications can be prevented. Studies showed that intense preoperative smoking cessation programs, including nicotine replacement medication and individual counseling started at least four weeks before surgery, could benefit surgical patients by reducing their risk of developing complications following their surgery [9,10]. Smoking cessation has a clear advantage over continued smoking in terms of preventing postoperative problems. It has been demonstrated that the smoking cessation phase influences complication rates, with each week of smoking cessation reducing the rate of postoperative complications significantly [11]. A previous study by Theadom found that short-term smoking cessation before surgery may lower the incidence of postoperative complications compared to continuing to smoke [10]. This highlights the significance of assessing people's awareness of the risks of smoking and encouraging them to quit. A study conducted on surgery patients in Abha City, Saudi Arabia, regarding their awareness of the surgery-related adverse effects of smoking revealed that the patients had a high level of knowledge on this subject [12]. However, another study conducted among the general population in Al-Ahsa City, Saudi Arabia, reported a poor level of knowledge regarding the topic [13]. This emphasizes the lack of knowledge regarding the surgery-related adverse effects of cigarette smoking among the general population. Therefore, we aimed to evaluate the public's understanding of how smoking can negatively impact surgical outcomes in the Makkah region. This study will contribute to a broader assessment of national knowledge on this topic and reinforce the importance of smoking cessation for surgical patients.

## Materials and methods

This descriptive cross-sectional study used a self-administered electronic questionnaire distributed among adults in the Makkah region, Saudi Arabia, between the 8th of March 2024 and the 8th of April 2024. Data collection started after ethical approval was obtained from the Research Ethics Committee of the Faculty of Medicine, Umm Al Qura University, Makkah, Kingdom of Saudi Arabia (Ethical Approval Number HAPO-02-K-012-2024-04-2111).

The study population consisted of adults over the age of 18 from the Makkah region in Saudi Arabia. Healthcare providers were excluded. The sample size was calculated utilizing Epi Info™ 7.1.5 (Centers for Disease Control and Prevention, Atlanta, Georgia, USA). The minimum number of participants was 384, with a 5% margin of error and a 95% confidence level; convenience sampling methods were used for data collection. The following formula was applied:

$\text {Sample size} (n) = \frac{\mathrm{DEFF} \times N\times p (1-p)}{\left( \frac{d^2}{Z^2_{(1-\alpha/2)}} \times (N-1) \right) + p(1-p)}$,

where n indicates the sample size, N is the study population of Makkah City (approximately 2,184,560, General Authority for Statistics KSA, 2022), and p is the most significant percentage of any community's properties that were surveyed, which was assumed to be 50%. The hypothesized percentage of the specific outcome frequency in the population (p) may be counted as follows: 50% ± 5. Confidence limits as a percentage of 100 (absolute ±) (d) were set at 5%, and the design effect for cluster surveys (DEFF) was set at number one.

Using this formula, the required sample size was determined, resulting in a sample of 385 participants. Future research requires a larger sample size to compensate for possible data loss.

The tool used in this study to evaluate the participants' awareness and knowledge of the surgery-related adverse effects of cigarette smoking was a validated questionnaire from a previous study [13] designed to assess participants' understanding of the link between smoking and surgical complications. The questionnaire included sections on personal information, smoking habits, knowledge of smoking and surgery, awareness of smoking health risks, and knowledge of smoking surgical complications. An online Arabic version of the questionnaire was created using Google Forms and shared on social media. Before starting the questionnaire, participants were asked to provide their consent and were given the researcher's contact information in case they had any questions.

After data collection, the dataset was reviewed for completeness and accuracy, and subsequently analyzed using the Statistical Package for the Social Sciences (SPSS), version 26 (IBM Corp., Armonk, New York, USA). Descriptive statistics were used to summarize participant characteristics, including gender, age, education level, and smoking habits. Chi-square tests and odds ratios (OR) were applied to assess the relationships between these factors and knowledge of smoking-related health risks and surgical complications. Smoking habits were summarized using frequencies and percentages, including smoking status (non-smoker, current smoker, former smoker), duration of smoking, and number of cigarettes smoked per day among current smokers, as well as time since quitting for ex-smokers. Awareness and sources of information about smoking's effects on surgery were presented as percentages, highlighting the most trusted sources. Multivariate logistic regression was applied to identify factors associated with knowledge, adjusting for potential confounders. The analysis included gender, smoking status, previous surgical experience, and whether participants received advice from a doctor to quit smoking prior to surgery. A p-value of less than 0.05 was considered statistically significant.

## Results

A total of 447 individuals initially agreed to take part in the study, but 43 were excluded for not meeting the inclusion criteria, leaving 404 in the final analysis. Of these, 206 (51%) were female and 198 (49%) were male. With respect to age, 163 (40.3%) were between 18 and 21 years old, while the majority, 183 (45.3%), were aged 22-30 years. Only 16 respondents (4%) were between 31 and 40 years, and 42 (10.4%) were aged 41 or older. Regarding education, 22 members of the sample (5.4%) had higher education, 307 (75.6%) had university-level education, and 79 (19.6%) had secondary or lower education. Most of the cohort reported a monthly income of ≤5000, with 287 (71%) falling into this category. Additionally, 307 (75.6%) were students (Table 1).

**Table 1:** Sociodemographic characteristics of participants.

Characteristics	Number	%
Gender		
Male	198	49.0
Female	206	51.0
Age (years)		
18-21	163	40.3
22-30	183	45.3
31-40	16	4.0
41 or more	42	10.4
Marital status		
Single	326	80.7
Married	77	19.1
Divorced or widowed	1	0.2
Income		
<5000	287	71.0
5000-10,000	61	15.1
10,001-20,000	37	9.2
>20,000	19	4.7
Occupation		
Student	307	75.6
Unemployed	24	5.9
Employed	70	17.3
Retired	3	0.7
Level of education		
Below secondary (primary or intermediate)	4	1.0
Secondary	75	18.6
University	303	75.0
Higher education	22	5.4

Table 2 summarizes participants' smoking behavior, exposure to secondhand smoke, and awareness of smoking-related surgical risks. Most participants (84.9%) were non-smokers, while 9.7% were current smokers and 5.4% were former smokers. Among current smokers, the majority had been smoking for less than five years (43.6%), and a significant portion smoked fewer than five cigarettes a day (41%). Most ex-smokers had quit within the last five years (81.8%). In terms of exposure, 25.5% of participants lived with a smoker, and 34.6% of those reported smoking indoors. Additionally, 54.7% were exposed to smokers outside the home, with 12.1% experiencing daily exposure. Regarding awareness of smoking's effects on surgery, 63.9% of participants had heard about it, with the majority learning about the risks through healthcare providers' social media posts (68.3%). However, among smokers who had surgery, only 22% were advised by their doctor to quit, and 31.4% actually quit before the procedure. The primary reason for quitting was a doctor's recommendation (52.2%).

**Table 2:** Smoking habits, exposure, and awareness of surgical adverse effects among participants.

Smoking data		No	%
Are you a smoker?	No	343	84.9%
	Yes	39	9.7%
	Ex-smoker	22	5.4%
If you are a smoker, for how long have you been smoking? (years)	Less than 5 years	17	43.6%
	5-10 years	14	35.9%
	More than 10 years	8	20.5%
If you are a smoker, how many cigarettes do you smoke per day? (cigarettes)	Less than 5	16	41.0%
	5-10	14	35.9%
	11-20	6	15.4%
	More than 20	3	7.7%
If you used to smoke but you have quit, for how long did you quit? (years)	Less than 5 years	18	81.8%
	5-10	2	9.1%
	More than 10 years	2	9.1%
Do you live with a smoker in the same house?	No	301	74.5%
	Yes	103	25.5%
Where did he/she smoke?	Smoke inside house	71	34.6%
	Smoke outside house	134	65.4%
Are you exposed to a smoker outside the house?	No	183	45.3%
	Yes	221	54.7%
Usually, how long are you usually exposed to a smoker inside or outside the house (days per week)?	Less than 2	300	74.3%
	3-6	55	13.6%
	Everyday	49	12.1%
Where did he/she smoke?	Smoke inside house	71	34.6%
	Smoke outside house	134	65.4%
Are you exposed to a smoker outside the house?	No	183	45.3%
	Yes	221	54.7%
Usually, how long are you typically exposed to a smoker inside or outside the house (days per week)?	Less than 2	300	74.3%
	3-6	55	13.6%
	Everyday	49	12.1%
Have you ever done a surgery? or any surgical procedure?	No	297	73.5%
	Yes	107	26.5%
If you are a smoker, and you have undergone surgery, did your treating doctor ask you to stop smoking a few days before surgery?	No	85	78.0%
	Yes	24	22.0%
If you are a smoker, and you have undergone surgery, did you stop smoking before surgery?	No	70	68.6%
	Yes	32	31.4%
If the answer is 1, why did you stop smoking before the surgery?	I already knew the surgical adverse effects of smoking	32	47.8%
	My doctor advised me to stop	35	52.2%
Have you heard about surgical adverse effects due to cigarette smoking?	No	146	36.1%
	Yes	258	63.9%
If you have heard about surgical adverse from social media, do you follow healthcare providers' accounts?	No	128	31.7%
	Yes	276	68.3%

Our results suggest that 258 (63.9%) of the participants had heard about the surgical adverse effects caused by cigarette smoking. Of the smokers who underwent surgery, only 24 (22%) were advised by their doctor to stop smoking a few days prior to the surgery, and nearly 8% stopped smoking before the surgery. Of the participants who stopped smoking before surgery, 35 (52.2%) reported that they did so because their doctor advised them to stop, while 32 (47.8%) reported that they did so because they were already aware of the surgery-related adverse effects of smoking (Table 3).

**Table 3:** Smoking and surgical procedure data.

Smoking and surgical procedures data	n (%)
Have you ever done a surgery? or any surgical procedure	
Yes	107 (26.5)
No	297 (73.5)
If you are a smoker, and you have undergone a surgery, did your treating doctor ask you to stop smoking a few days prior to surgery?	
Yes	24 (22.0)
No	85 (78.0)
If you are a smoker, and you have undergone a surgery, did you stop smoking before the surgery?	
Yes	32 (7.9)
No	70 (68.6)
Why did you stop smoking before the surgery?	
I already knew the surgical adverse effects of smoking	32 (47.8)
My doctor advised me to stop	35 (52.2)
Have you heard about surgical adverse effects due to cigarette smoking?	
Yes	258 (63.9)
No	146 (36.1)
If you have heard about surgical adverse from social media, do you follow healthcare providers’ accounts?	
Yes	276 (68.3)
No	128 (31.7)

Table 4 illustrates respondents' awareness and perceptions of smoking-related health and surgery risks. Regarding health risks, the majority of participants were aware of several smoking-related conditions. Lung cancer was the most recognized, with 93.3% of participants reported for the risk, followed by coronary heart disease and hypertension (81.4% and 79.7% of respondents, respectively). Premature aging and sexual problems were known by 78.2% and 77.2%, while awareness of increased carbon monoxide levels (73.8%), sensory problems (72.8%), and peptic ulcers (73.5%) was also high, though a notable portion of respondents expressed uncertainty (22%-24%). When it comes to surgery risks, a similar pattern emerged. Most respondents were aware of the increased risk of future heart or lung problems (92.1%), and a majority recognized slower wound healing (69.1%) and increased hospital admission period (76.5%). However, fewer respondents were aware of risks such as increased infection (64.9%) and anesthetic complications (66.8%), with a significant portion unsure about these risks (30.7%-30.4%). Regarding smoking cessation before surgery, nearly half of the participants (48.3%) agreed that the ideal cessation period is four to six weeks, though 47.5% were unsure. Furthermore, 84.2% of respondents believed it was necessary to quit smoking after surgery as well, with only 14.4% expressing uncertainty.

**Table 4:** Awareness and perceptions of smoking-related health and surgery risks among respondents.

Effect	No		Yes		I don't know	
	No	%	No	%	No	%
Smoking-related health risks						
Increased carbon monoxide level	9	2.2%	298	73.8%	97	24.0%
Sensory problems	18	4.5%	294	72.8%	92	22.8%
Premature aging	19	4.7%	316	78.2%	69	17.1%
Sexual problems	17	4.2%	312	77.2%	75	18.6%
Peptic ulcer	20	5.0%	297	73.5%	87	21.5%
Hypertension	13	3.2%	322	79.7%	69	17.1%
Coronary heart disease	6	1.5%	329	81.4%	69	17.1%
Lung cancer	5	1.2%	377	93.3%	22	5.4%
Esophageal cancer	12	3.0%	330	81.7%	62	15.3%
Smoking-related surgery risks						
Slower healing of wounds after surgery	15	3.7%	279	69.1%	110	27.2%
Increased risk of infection after surgery	18	4.5%	262	64.9%	124	30.7%
Increased complications with anesthetic	11	2.7%	270	66.8%	123	30.4%
Increased risk of future heart or lung problems	4	1.0%	372	92.1%	28	6.9%
Increased hospital admission period	15	3.7%	309	76.5%	80	19.8%
The ideal smoking cessation period before surgery is four to six weeks	17	4.2%	195	48.3%	192	47.5%
Do you think it is necessary to stop smoking after surgery (in addition to preoperative cessation)?	6	1.5%	340	84.2%	58	14.4%

Table 5 shows the association between demographic/behavioral factors and knowledge scores on smoking health risks and surgical complications. Gender does not significantly influence knowledge, as indicated by the odds ratio (OR) for both health risks (OR = 0.958, 95% CI: 0.501-1.833, p = 0.897) or surgical complications (OR = 0.673, 95% CI: 0.421-1.075, p = 0.097). Smoking status is significantly associated with knowledge of smoking health risks (p = 0.015, OR = 0.850, 95% CI: 0.809-0.894). However, no significant association is observed between smoking status and knowledge of surgical complications (p = 0.245, OR = 1.661, 95% CI: 0.701-3.933). Previous surgical experience and doctor advice to quit smoking prior to surgery do not show significant associations with either knowledge domain. While doctor advice shows a higher OR for surgical complication knowledge (OR = 2.00, 95% CI: 0.611-6.54), the result is not statistically significant (p = 0.246).

**Table 5:** Relation between knowledge assessment scores and biodemographic/behavioral factors. *P-value <0.05; ORA: adjusted odds ratio.

Biodemographic/behavioral factors	People with high smoking health risk score			People with high smoking surgical complications score		
	P-value	ORA	95% CI for OR (lower–upper)	P-value	ORA	95% CI for OR (lower–upper)
Gender						
Female versus male	0.897	0.958	0.501–1.833	0.097	0.673	0.421–1.075
Smoking status						
Smoker versus non-smoker	0.015*	0.850	0.809–0.894	0.245	1.661	0.701–3.933
Have undergone surgery before						
Yes versus no	0.549	0.802	0.389–1.652	0.632	0.881	0.525–1.479
Doctor advised ceasing smoking prior to surgery						
Yes versus no	0.849	1.172	0.227–6.050	0.246	2.00	0.611–6.543

**Figure 1:**
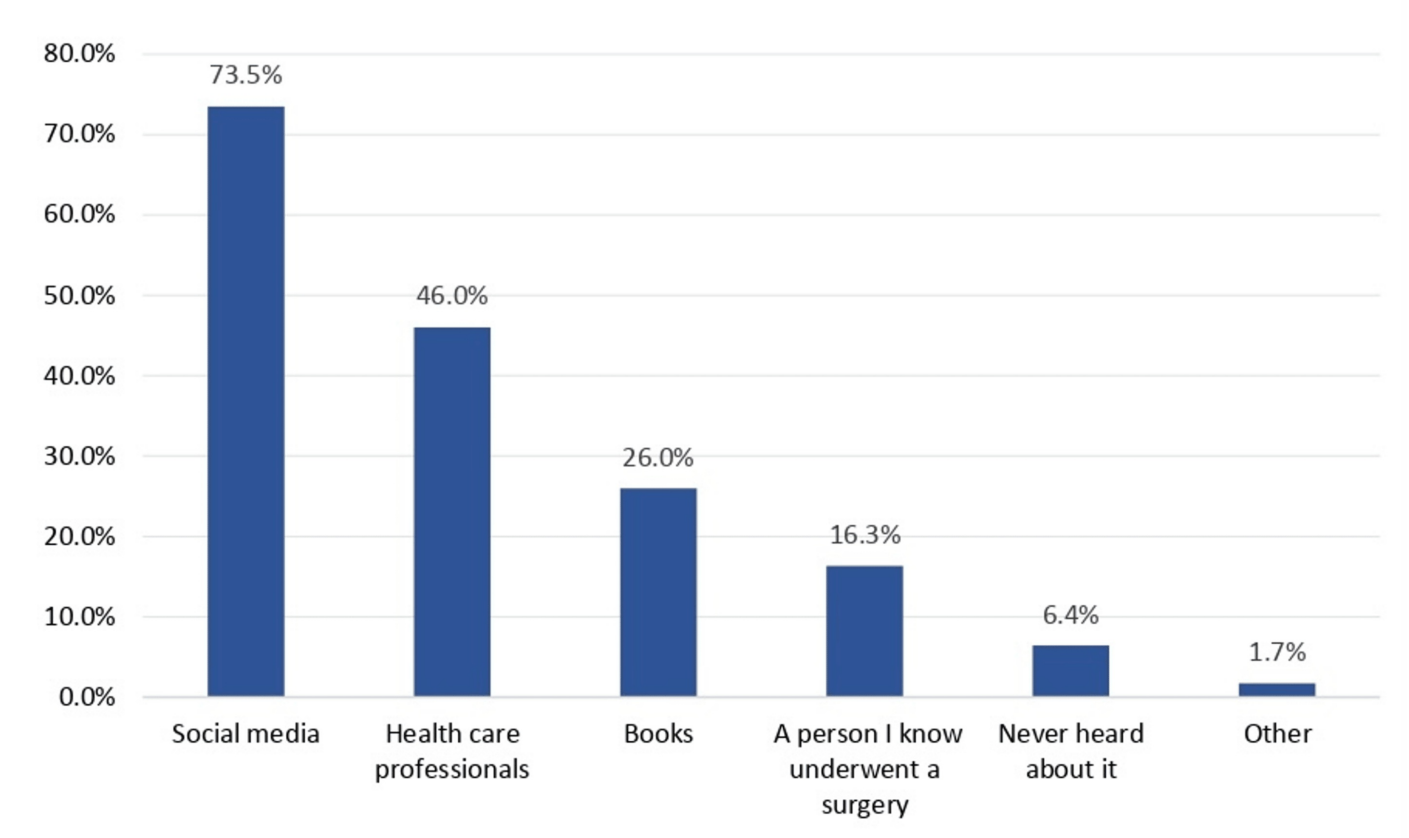
The source of information about surgical adverse effects due to cigarette smoking.

Figure 1 shows the source of information about surgical adverse effects due to cigarette smoking. Social media was the most common source, with 73.5% of participants indicating they had learned about the surgical risks of smoking through this platform. This was followed by Books (26.0%); healthcare professionals provided information to 46.0% of participants, and 54.0% did not learn from this source. Personal experience, such as knowing someone who underwent surgery, was reported by 16.3% of participants. Lastly, 93.6% of participants had never heard about the adverse effects of smoking on surgery. As for the most trusted sources of information, the most reported source included "Health care professionals" at 74.0%, followed by "Medical websites" at 66.1%. "Books" rank third with 38.9%, while "Social media" accounts for 28.7%. "A person I know underwent a surgery" follows at 22.3%, and "Internet" slightly exceeds "Documents," with 19.3% and 14.1%, respectively.

## Discussion

The objective of this study was to obtain insight into perceptions and knowledge regarding surgery-related adverse effects caused by cigarette smoking among the adult population of the Makkah region in Saudi Arabia. The prevalence of smoking in our study was 9.7%, and 84.9% of participants reported that they were non-smokers. The global prevalence of smoking among adults is 32.6% (ranging from 32.2% to 33.1%) for men and 6.5% (ranging from 6.3% to 6.7%) for women [14].

The overall prevalence of smoking in the Middle East is 34.7% at the time of this study, with notably higher rates among males than females at 42.9% and 27.5%, respectively [14]. The lower smoking prevalence observed in this study may be explained by the predominance of young university participants and the use of social media-based recruitment, which likely limited the inclusion of older or habitual smokers.

The current study reported that the most frequently recognized health risk associated with smoking was lung cancer, followed by esophageal cancer (81.7%) and coronary artery disease (recognized by 93.3%, 81.7%, and 81.4% of participants, respectively). Sensory problems were the least frequently recognized health risk associated with smoking (72.80%). These results are similar to those of a previous study, wherein 44.1% of the students surveyed reported awareness of lung cancer, and 10.4% mentioned heart disease and stroke [15]. A prior study conducted in China among male smokers reported that smoking leads to lung disease, oral cancer, heart disease, and strokes [16].

When considering surgical complications, 92.1% of participants agreed that smoking could elevate the risk of future heart or lung issues, 76.5% acknowledged that smoking could lead to more hospital admissions, and 66.8% recognized an increased risk of complications related to anesthesia. This finding aligns with a previous study conducted in China [17], which revealed that 67%, 33.2%, and 29.5% of participants believed smoking could lead to lung cancer, heart attacks, and strokes, respectively. Additionally, 26.0% of participants reported that they thought smoking could cause all of these conditions. Similar results were shown in a study [18] conducted in Jordan, in which 90.2% of participants identified a relationship between smoking and an increased risk of cardiovascular disease, and 94% were aware of the association between smoking and respiratory illnesses.

Our study demonstrates that the majority of participants (93.6%) were aware of the adverse effects of smoking cigarettes on the ability to heal after surgery. Social media and healthcare professionals were the primary sources of information (for 73.5% and 46% of participants, respectively). Medical professionals were considered the most reliable source of medical information by 74% of participants, followed by medical websites (66.1%). Similar results were shown in a study performed in Alhasa [19], revealing that only 32.9% of participants were aware of the surgical adverse effects linked to cigarette smoking. Less than half of respondents (43.2%) got their knowledge about these effects from social media, while just 28.1% received this information from a healthcare provider. Additionally, approximately 49.4% considered healthcare professionals to be one of the most reliable sources for information on the surgery-related adverse effects associated with cigarette smoking. This demonstrates how crucial it is for doctors to advise and motivate their patients to give up smoking before surgery.

Our study found a significant correlation (P = 0.015) between smoking status and knowledge regarding smoking health risks. However, contrary to our findings, another study found a statistically significant association between knowledge of the health effects of smoking and smoking status. It revealed that 62.5% of smokers had poor knowledge, and only 37.5% of adolescents with good knowledge reported ever having smoked a cigarette [15]. This discrepancy may reflect differences in sample composition and context; our study included educated adults, whereas the comparative study focused on adolescents with lower health literacy and limited exposure to health education. Variations in age, education level, and access to public health information likely influenced the divergent findings. Xu et al. found that 62.5% of smokers had poor knowledge, and only 37.5% of adolescents with good knowledge reported ever having smoked a cigarette [16]. A study conducted in Japan found that there is a significant correlation between smoking awareness and educational attainment, suggesting that higher education levels often lead to increased awareness of the health risks associated with smoking [19]. 

When compared with studies conducted in Abha and Al-Ahsa [12,13], several contextual, demographic, and methodological factors may explain the observed variations in findings. The current study primarily included young adults, with over 85% of participants aged below 30 years and more than three-quarters being university students, whereas the Abha study targeted surgical patients, most of whom were middle-aged individuals already engaged with healthcare services [12]. This difference in population characteristics may account for the higher levels of awareness reported in Abha, as surgical patients are more likely to have received preoperative counseling or medical advice regarding the adverse effects of smoking on surgical outcomes. In contrast, our younger, community-based cohort may have had less direct exposure to such information. Similarly, the Al-Ahsa study involved a broader community sample with a higher proportion of employed and married individuals, demographic groups that often demonstrate distinct health-seeking behaviors and greater health literacy compared with university-aged participants in Makkah [13].

In addition to these contextual differences, methodological variations may have contributed to the discrepancies among studies. The Abha study focused exclusively on smokers undergoing surgery, whereas the present research included both smokers and non-smokers from the general adult population, which could have resulted in a lower baseline level of awareness regarding smoking-related surgical complications. Furthermore, cultural and regional distinctions between Makkah, a metropolitan, transient, and diverse population, and the more localized settings of Abha and Al-Ahsa may have influenced participants' exposure to public health campaigns and physician counseling. Variations in education levels, data collection periods, and survey instrument design may also have affected the comparative outcomes observed across these studies.

These findings imply that there is a lack of knowledge on the adverse effects of smoking after surgery. For this reason, we advise establishing and implementing a robust program for preoperative smoking cessation in order to give smokers the necessary information and educational resources, encourage them to stop, and provide referrals for behavioral modification.

The strengths of this study include a good sample size. For instance, the sample size indicates a good response rate. Furthermore, this is the first broad study conducted in the Makkah region. However, our study also has some limitations. First, some participants may not have had much experience with people who had surgery after smoking. This could have affected their opinions. Furthermore, as it was cross-sectional, the study could not establish causal relationships. Convenience sampling via social media may have excluded older adults or individuals with limited digital literacy, introducing potential selection bias. Additionally, reliance on self-reported data may have resulted in recall bias. Nonetheless, the findings provide important insight into public awareness of smoking-related health and surgical risks in the Makkah region.

## Conclusions

This study reveals a significant gap in knowledge specifically related to the adverse effects of cigarette smoking on surgical outcomes among adults in the Makkah region. While general awareness of smoking's health hazards was relatively high, participants demonstrated limited understanding of its impact on postoperative complications. Addressing this gap requires comprehensive smoking cessation initiatives that incorporate targeted educational campaigns, preoperative counseling, access to nicotine replacement therapies, and structured follow-up support tailored to the Makkah population.

## Appendices

Appendix A: Questionnaire

Do you consent to participate in this study?

A. Yes

B. No

Biodemographic data:

Age (years):

A. <18

B. 18-21

C. 22-30

D. 31-40

E. 41 or more

Gender:

A. Female

B. Male

Marital status:

A. Married

B. Single

C. Others

What is your nationality?

A. Saudi

B. non-Saudi

What is your place of residence?

A. Makkah

B. Jeddah

C. Taif

D. Al-Jamoum

E. Al-Qunfudhah

F. Other

Income:

A. <5000

B. 5000-10,000

C. 10,001-20,000

D. >20,000

Education level:

A. Able to read

B. Elementary

C. Intermediate

D. Secondary

E. University

Smoking habit

Are you smoker?

A. Yes

B. No

C. Ex-smoker

If you are a smoker, for how long have you been smoking? (years)

A. Less than 5 years

B. 5-10 years

C. More than 10 years

If you are a smoker, how many cigarettes do you smoker per day (cigarettes)

A. Less than 5

B. 5-10

C. 11-20

D. More than 20

If you used to smoke but you have quitted, for how long did you quit? (years)

A. Less than 5 years

B. 5-10 years

C. More than 10 years

Do you live with a smoker at the same house?

A. Yes

B. No

Where did he/she smoke?

A. Smoke inside house

B. Smoke outside house

Do you expose to another smoker outside house?

A. Yes

B. No

Usually, how long do you expose to another smoker inside or outside house (days per week)?

A. Less than 2

B. 3-6

C. Everyday

How long do you expose to other smokers inside or outside the house (hours per day)

A. Less than 1

B. 1-3

C. More than 3

Knowledge assessment for smoking with surgical procedures

Have you ever done a surgery? Or any surgical procedure?

A. Yes

B. No

If you are a smoker, and you have undergone a surgery, did your treating doctor ask you to stop smoking few days prior to surgery?

A. Yes

B. No

If you are a smoker, and you have undergone a surgery, did you stop smoking before surgery?

A. Yes

B. No

If the answer is yes, why did you stop smoking before the surgery?

A. I already knew the surgical adverse effects of smoking

B. My doctor advised me to stop

Have you heard about surgical adverse effects due to cigarette smoking?

A. Yes

B. No

If you have heard about surgical adverse from social media, do you follow health-care providers' accounts?

A. Yes

B. No

Determine the source that you have heard about surgical adverse effects due to cigarette smoking:

A. Social media

B. Health care professionals

C. A person I know underwent a surgery

D. Books

E. Others

What are the most trusted sources of information for surgery-related adverse effects of cigarette smoking?

A. Social media

B. Health care professionals

C. A person I know underwent a surgery

D. Medical websites

E. Internet

F. Documents

G. Books

Knowledge assessment for smoking health risk

Increased carbon monoxide level

A. Yes

B. No

C. I do not know

Sensory problems

A. Yes

B. No

C. I do not know

Premature aging

A. Yes

B. No

C. I do not know

Sexual problems

A. Yes

B. No

C. I do not know

Peptic ulcer

A. Yes

B. No

C. I do not know

Hypertension

A. Yes

B. No

C. I do not know

Coronary heart disease

A. Yes

B. No

C. I do not know

Lung cancer

A. Yes

B. No

C. I do not know

Esophageal cancer

A. Yes

B. No

C. I do not know

Knowledge assessment for smoking surgical complications 

Smoking can cause:

Slower healing of wounds after surgery

A. Yes

B. No

C. I do not know

Increased risk of infection after surgery

A. Yes

B. No

C. I do not know

Increased pain after surgery

A. Yes

B. No

C. I do not know

Increased complications with anesthetic

A. Yes

B. No

C. I do not know

Increased risk of future heart or lung problems

A. Yes

B. No

C. I do not know

Increased hospital admission period

A. Yes

B. No

C. I do not know

The ideal smoking cessation period before surgery is 4-6 weeks

A. Yes

B. No

C. I do not know

Do you think it is necessary to stop smoking after surgery (in addition to preoperative cessation)?

A. Yes

B. No

C. I do not know
